# Nanoparticulated Honokiol Mitigates Cisplatin-Induced Chronic Kidney Injury by Maintaining Mitochondria Antioxidant Capacity and Reducing Caspase 3-Associated Cellular Apoptosis

**DOI:** 10.3390/antiox8100466

**Published:** 2019-10-09

**Authors:** Hung-Ting Liu, Tse-En Wang, Yu-Ting Hsu, Chi-Chung Chou, Kai-Hung Huang, Cheng-Chih Hsu, Hong-Jen Liang, Hui-Wen Chang, Tzong-Huei Lee, Pei-Shiue Tsai

**Affiliations:** 1 Department of Veterinary Medicine, School of Veterinary Medicine, National Taiwan University, Taipei 10617, Taiwan; r04629005@ntu.edu.tw (H.-T.L.); f05629003@ntu.edu.tw (T.-E.W.); r07629002@ntu.edu.tw (Y.-T.H.); huiwenchang@ntu.edu.tw (H.-W.C.); 2 Graduate Institute of Veterinary Medicine, School of Veterinary Medicine, National Taiwan University, Taipei 10617, Taiwan; 3 Department of Veterinary Medicine, College of Veterinary Medicine, National Chung-Hsing University, 402 Taichung, Taiwan; ccchou@nchu.edu.tw; 4 Department of Chemistry, National Taiwan University, Taipei 10617, Taiwan; r05223167@ntu.edu.tw (K.-H.H.); ccrhsu@ntu.edu.tw (C.-C.H.); 5 Department of Food Science, Yuanpei University, 30015 Hsinchu, Taiwan; jenliang@mail.ypu.edu.tw; 6 Graduate Institute of Molecular and Comparative Pathobiology, School of Veterinary Medicine, National Taiwan University, Taipei 10617, Taiwan; 7 Institute of Fisheries Science, National Taiwan University, Taipei 10617, Taiwan; thlee1@ntu.edu.tw; 8 Research Center for Developmental Biology and Regenerative Medicine, National Taiwan University, Taipei 10617, Taiwan

**Keywords:** honokiol, cisplatin, kidney injury, oxidative stress, apoptosis, nanotechnology

## Abstract

Cisplatin is a potent anti-cancer drug, however, its accompanied organ-toxicity hampers its clinical applications. Cisplatin-associated kidney injury is known to result from its accumulation in the renal tubule with excessive generation of reactive oxygen species. In this study, we encapsulated honokiol, a natural lipophilic polyphenol constituent extracted from *Magnolia officinalis* into nano-sized liposomes (nanosome honokiol) and examined the in vivo countering effects on cisplatin-induced renal injury. We observed that 5 mg/kg body weight. nanosome honokiol was the lowest effective dosage to efficiently restore renal functions of cisplatin-treated animals. The improvement is likely due the maintenance of cellular localization of cytochrome c and thus preserves mitochondria integrity and their redox activity, which as a consequence, reduced cellular oxidative stress and caspase 3-associated apoptosis. These improvements at the cellular level are later reflected on the observed reduction of kidney inflammation and fibrosis. In agreement with our earlier in vitro study showing protective effects of honokiol on kidney cell lines, we demonstrated further in the current study, that nanosuspension-formulated honokiol provides protective effects against cisplatin-induced chronic kidney damages in vivo. Our findings not only benefit cisplatin-receiving patients with reduced renal side effects, but also provide potential alternative and synergic solutions to improve clinical safety and efficacy of cisplatin treatment on cancer patients.

## 1. Introduction

Cisplatin (cis-diamminedichloroplatimum II), a platinum-containing compound has been used as an anti-cancer drug since the 1970s [1]. One of the therapeutic effects comes from its ability to form inter- and intra-strand DNA adducts in highly proliferating tumor cells and leads to cell necrosis and/or apoptosis [2,3]. Although cisplatin is one of the most effective therapeutic anti-cancer compounds in human and veterinary medicine, its accompanied renal toxicity often restrains its systemic application [4,5,6]. Clinical oncologists tend to reduce cisplatin-induced organ toxicity by replacing cisplatin with carboplatin or oxaliplatin or by applying cisplatin topically rather than systemic administration. However, these alternative compounds and approaches greatly reduce the in vivo efficacy for cancer treatments and often result in other unexpected side effects. One of the cisplatin-associated nephrotoxicities is resulted from its preferential accumulation in the renal tubules due to imbalanced uptake and output of cisplatin by protein transporters [7,8,9]. Apart from cisplatin’s effect on DNA, cisplatin is also known to interfere in the electron transport system of the mitochondria and thus, enhances the generation of reactive oxygen species (ROS), nitrogen species (RNS) that subsequently lead to mitochondrial dysfunction [10]. Healthy individuals possess antioxidant defense mechanisms that facilitate the quenching of ROS/RNS and maintain the equilibrium between pro- and anti-oxidants, however, under pathological conditions, the uncontrolled production of endogenous ROS/RNS exceeds cellular antioxidant capability, which in consequence, results in oxidative stress (OS) and the activation of intrinsic apoptosis cascade [10,11].

Honokiol (HNK), a polyphenol natural constituent from *Magnolia officinalis* has been demonstrated to have many bioactivities, such as anti-allergy [12], anti-anxiety, anti-depression [13], anti-cancer [14], anti-inflammation [12,15] and neuroprotection [16]. Recent in vitro studies including ours demonstrated that HNK exhibits antioxidant properties via its ability to reduce cellular ROS production and thus maintained cellular redox balance [17,18,19]. Although HNK is proven to be a multifunctional small molecule, its low aqueous solubility often hampers its bioavailability. To overcome this natural chemical solubility barrier of HNK, recent publications proposed that nanotechnology might be a promising strategy to enhance the solubility and stability of phytochemicals and to prolong in vivo half-life of lipophilic compounds by avoiding high levels of degradation during administration [20]. Earlier reports demonstrated that nanosuspension containing HNK can alter the bio-distribution of HNK with increased tissue bioavailability and serum concentration [20,21]. This approach allows slow release of HNK in the body via systemic administration, which prolongs its antioxidant effects in vivo. Based on the above-mentioned evidence, HNK formulated in nanosuspension (nanosome HNK, hereafter termed “nHNK”) is a promising approach to be exploited for the attenuation of cisplatin-induced renal toxicity and to improve clinical safety of cisplatin in cancer patients. In our earlier study, we demonstrated in vitro that HNK protects against cisplatin-induced renal damages by maintaining cellular localization of E-Cadherin and Occludin, promoting the polymerization of actin and tubulin cytoskeleton and counteracting cisplatin-induced oxidative stress. In this study, we apply a nano-sized liposome preparation procedure using ultra high-pressure homogenization with a minimal amount of organic solvent to produce nHNK, and extend our earlier findings toward an in vivo model system to evaluate whether application of nHNK by intravenous tail-vein injection could effectively attenuate cisplatin-induced kidney injury, as we observed earlier in our in vitro cell-based study [19]. This study will not only validate our earlier in vitro study but will also potentially improve clinical safety of cisplatin that allows applications of nanotechnology to encapsulate HNK in nanosuspension formulation to increase its tissue bioavailability with prolonged half-life in vivo.

## 2. Materials and Methods

### 2.1. Chemicals, Reagents, Antibodies 

Unless otherwise stated, reagents and chemicals were obtained from Sigma Aldrich (St. Louis, MO, USA), including anti-cancer compound Cis-Diamineplatinum(II) dichloride (Cisplatin, Cat. #479306, purity ≥ 99.9%) and α-Tocopherol (Vitamin E, Cat.#T3251, purity > 96%). 2-(4-hydroxy-3-prop-2-enyl-phenyl)-4-prop-2-enyl-phenol (Honokiol, Cat. #SLK S2310, purity: 99.81%) was obtained from Selleckchem (Houston, TX, USA). Rabbit polyclonal anti-tumor necrosis factor-α (TNF-α, #Ab6671), anti-cytochrome c (#Ab90592) antibodies, mouse monoclonal anti-8-Hydroxydeoxyguanosine (8-OHdG, #Ab62623), anti-β actin (#Ab8226), and anti-glyceraldehyde 3 phosphate dehydrogenase (GADPH, #Ab9484) were obtained from Abcam (Cambridge, UK). Mouse monoclonal anti-caspase 6 (#SC377393) and rabbit polyclonal anti-caspase 3 (#SC9665) were obtained from Santa Cruz (Dallas, TX, USA) and Cell Signaling Technology (Danvers, MA, USA), respectively. DeadEnd Fluorometric Terminal Deoxynucleotidyl Transferase dUTP Nick End Labeling (TUNEL) assay kit was acquired from Promega (Madison, WI, USA). All secondary antibodies were purchased from Jackson ImmunoResearch Laboratories Inc. (West Grove, PA, USA).

### 2.2. Cisplatin-Induced Chronic Kidney Injury Mouse Model

Animal experiments were approved and carried out under the regulation and permission of institutional animal care and use committee (IACUC) protocols at National Taiwan University (Taiwan, NTU-103-EL-37). Twelve-week-old male institute cancer research (ICR) mice (obtained from National Laboratory Animal Center, Taiwan) were housed individually in the metabolic cages for at least 5 days prior to the experiments. Liposome-encapsulated HNK was prepared and provided by Dr. Liang (Yuanpei University, Taiwan), qualitative and quantitative characterizations of prepared nHNK were carried out at the department of chemistry at National Taiwan University (NTU Mass Spectrometry Platform) and results are shown in the supplementary materials (Figure S1). From our analytic data of nHNK, honokiol encapsulating efficiency in the nano-sized liposome was 58.1% ± 2.44% (Figure S1). To establish cisplatin-induced chronic kidney injury and treatment protocols, an initial dosage-dependent (0, 1, 2.5, 5 mg/ kg B.W. nHNK) test was performed (*n* = 5 in each group) and evaluated based on parameters related to kidney physiology. After the lowest effective dosage was determined, a large-scale in vivo experiment (*n* = 10 in each group) was conducted by the chosen nHNK dosage. For the large-scale in vivo experiment, mice were randomly allocated into 4 experimental groups, as summarized in the supplementary materials (Figure S2) and described as follow: G1 (vehicle control): control mice received 100 μL of sterilized phosphate-buffered saline (PBS, vehicle control for cisplatin, red open circle) at week 0 and week 1. An additional 100 μL of empty nanosomes (vehicle control for HNK, blue open circle) was given to these animals via tail-vein injection (IV) at a three times/week interval for 6 weeks. G2 (nHNK group, nanosome HNK alone): mice in this group received 100 μL sterilized PBS at week 0 and week 1 and 5 mg/kg B.W. nHNK (blue closed circle, 100 μL volume) at a three-times/week interval. G3 (Cisplatin injury group): the kidney injury mouse model was established by 2 injections of cisplatin (10 mg/kg B.W., intraperinatal) at week 0 and week 1 (red closed circle), and animals in this group received an additional 100 μL of empty nanosomes at the same interval as mice in G2. G4 (Cisplatin/nHNK, treatment group): mice in the treatment group received 2 doses of cisplatin injections at week 0 and week 1, as in G3, and received 5 mg/kg B.W. nHNK, the same as mice in G2. 

### 2.3. Physical Evaluation, Serum and Urine Analyses

To evaluate the physiological condition of animals, body weight was measured and recorded bi-daily before compound injections and blood collection. Blood was collected weekly through orbital sinus using a heparin-coated capillary tube (Thermo Fisher Scientific, Waltham, MA, USA). Serum was separated from red blood cells by centrifugation at 2000× *g* for 10 min at 4 °C. Urine samples were collected weekly from the urine concentrating tube of the metabolic cages. Serum and urine samples were kept at 4 °C and submitted for measurements on the same day of collection. Blood urea nitrogen (BUN) and urine creatinine were analyzed using VITROS^®^ 350 Chemistry System (Ortho Clinical Diagnostics, Raritan, NJ, USA) at the Clinical Pathology Laboratory facility at the School of Veterinary Medicine, National Taiwan University Teaching Hospital. Serum indoxylsulfate (IS) was analyzed with high performance liquid chromatography (HPLC) (Hitachi F-1050 Fluorescence Spectrophotometer, Japan) at the School of Veterinary Medicine, National Chung-Hsing University (Taichung, Taiwan). Standardization of indoxylsulfate was carried out each time prior to the measurement, 0.5–10° ppm of purified indoxylsulfate (Sigma) was used to set up standardization points to build up the standardization curve (Figure S3). The HPLC mobile phase was sodium acetate buffer (pH 4.5) – acetonitrile (10:90, *v/v*). The excitation and emission wavelength were 280 nm and 375 nm, respectively. Triplicate injections (10 or 30 μL each) were performed for each sample using the auto-sampler with Mightysil RP-18 GP column (250 mmx 4.6 mm, 5 µm). HPLC was performed at a flow rate of 1 mL/min, and was monitored for 4 min after each injection, the IS peak was eluted at 2.0–2.4 min (Figure S3). Urine osmolality and protein concentration were measured with a freezing point Osmomat 3000 (Gonotec, Berlin, Germany) and a bicinchoninic acid (BCA) protein assay kit (Pierce, Wilton, IL, USA), respectively.

### 2.4. Kidney Morphology and Pathology Evaluations

After mice were euthanized, kidneys were first weighted before fixation and procedures for paraffin-embedding. Five μm kidney sections were deparaffinized in xylene, rehydrated and underwent antigen retrieval, as described earlier [22]. Sections were stained with hematoxylin and eosin (H and E) for general morphological evaluation or with Masson’s Trichrome stain to assess the level of fibrosis. Based on H and E-stained tissue sections, pathologic scoring was performed using the triple blind method by 3 licensed pathologists. Parameters including tissue inflammation, renal tubule lesions (i.e., atrophy, degeneration, necrosis, regeneration) and kidney cortical surface shrinkage were assessed and scored. The relative severity of each lesion was graded on a 0 to 3 scale and total pathologic scoring was calculated and expressed as the sum of all parameters. 

### 2.5. Cell Culture and Intracellular Reactive Oxygen Species (ROS) Production Measured by 2′, 7′-Dichlorofluorescein Diacetate

A stable cell line of Madin Darby Canine Kidney epithelial cell (MDCK) was acquired from American Type Culture Collection (ATCC, PTA-6500, Manassas, VA, USA). Cells were sub-cultured in Dulbecco’s Modification of Eagle’s Medium (DMEM, Gibco, Waltham, MA, USA) supplemented with 10% fetal bovine serum (FBS) and 1% penicillin-streptomycin-amphotericin B (Gibco) at 37.5 °C in a humidified atmosphere with 5% CO_2_. To compare the effects of different antioxidants (i.e., vitamin E and HNK) on the inhibition of ROS, cells were co-incubated with either vitamin E (1, 2.5, 5, 10 μM) or with HNK (1, 2.5, 5, 10 μM) in the presence of 10 μM cisplatin (for positive control). Cellular ROS was measured indirectly by membrane-permeable dye 2′, 7′-dichlorofluorescein diacetate (DCFH-DA, FL1-H) and cell viability was assessed simultaneously with propidium iodide (PI, FL2-H). After the required treatments, phenol red-free DMEM containing 25 μM DCFH-DA was used for further incubation at 37 °C for 30 min in the dark. Trypsinized MDCK cells were resuspended in PBS in the presence of 1 mg/mL PI for live/dead stain. After the staining, cells were washed, resuspended in ice-cold PBS and analyzed with FACScalibur flow cytometer (Becton and Dickinson, Pharmingen, Franklin Lakes, NJ, USA). 

### 2.6. Tissue Preparation and Immunohistochemistry Staining (IHC)

For immunohistochemistry staining (IHC), standard manufactory protocol was followed. In short, after the deparaffinization procedures, the slides were submerged in commercially available trilogy (Sigma) and heated to 121 °C for 3 min in autoclave and cool down to 45 °C for antigen retrieval. Endogenous peroxidase was removed by submerging the slides into 3% H_2_O_2_ (diluted with pure methanol). After blocking non-specific antigen by 5% normal goat serum (Jackson ImmunoResearch Laboratories Inc. PA, USA) for 1 h at room temperature (RT), anti-TNF-α antibody was applied to the tissues (1:200 diluted with blocking buffer) for overnight incubation at 4 °C to evaluate the presence of pro-inflammatory cytokine. The Leica Novolink Polymer Detection System (Leica, Solms, Germany) was used to generate positive signals following the manufacturer’s instruction. Tissues were subjected to hematoxylin counter stain and sealed with coverslips. For kidney fibrosis analysis, Masson’s trichrome stained sections were quantitatively assessed using the TissueFAXS PLUS system (TissueGnostic, Wien, Austria). 

### 2.7. Indirect Immunofluorescence (IFA) Staining and Image Acquisition

For indirect immunofluorescence staining, 5 μm paraffin-embedded tissue sections were deparaffinized, rehydrated and underwent antigen retrieval as mentioned above. After being blocked with 1% BSA for 30 min at RT, tissue sections were further permeabilized with 100% ice-cold methanol at −20 °C for 10 min. Anti-8-hydroxyguanosine (1:500), anti-caspase 3 (1:150) and anti-caspase 6 (1:150) antibodies were used for overnight incubation at 4 ºC. Sections were subsequently incubated with goat-anti-mouse/rabbit Alexa-594 (1:150 diluted with tri-based saline with tween 20 [TBST], 5 mM Tris, 250 mM sucrose, pH 7.4 with 0.05% *v/v* Tween-20) for 1.5 h at RT. Nuclei were counterstained with anti-fade mounting medium, Vectashield, in the presence of diamidino-2-phenylindole (DAPI, Vector Lab, Peterborough, UK). All samples were visualized under Olympus IX83 epifluorescent microscopy or with Leica TCS SP5 II confocal scanning microscopy. When necessary, all images in the same experiment were subtracted for background noise and contrast/brightness were adjusted to exactly the same extent. 

### 2.8. Antioxidation Ability Measurement

To evaluate nHNK effects on cellular redox status and mitochondria function, the OxiSelect^TM^ assay kit (Cell Biolabs, Inc., San Diego, CA, USA) was used to evaluate total antioxidant capacity (TAC) in the kidney samples. Kidney tissues were homogenized with homogenization buffer (250 mM sucrose, 1 mM ethylenediaminetetraacetic acid (EDTA), 20 mM Tris-Hepes, pH 7.5) and subsequently mixed with reaction buffer. Initiation and termination of reactions were followed by manufactory instruction. For kinetic measurement, sample mixtures were added into a 96-well microtiter plate and placed immediately into the SpextraMax M5 multiplate reader for optical density (OD) measurement for a consecutive 180 min at a 30 min interval at 490 nm (SpectraMax M5, Molecular Devices, San Jose, CA, USA). 

### 2.9. Separation of Mitochondrial- and Cytoplasm-Containing Fractions for Mitochondria Enzyme Status Assessment

Separation of mitochondria from the rest of the cytosolic proteins was carried out as previously described [23], with minor modifications. In brief, kidney tissues were rinsed with ice-cold DPBS and were sliced with a sterilized blade on ice. Sliced tissues were Dounce-homogenized on ice in the presence of ice-cold DPBS supplemented with protease inhibitor. Crude tissue homogenates were spun down at 1000× *g* for 3 min at 4 °C. Pellets were resuspended in ice-cold hypotonic CaRSB buffer (10 mM NaCl, 1.5 mM CaCl_2_, 10 mM Tris–hydrogen chloride, pH 7.5, supplemented with protease inhibitors) for 15 min before further centrifugation at 690× *g* for 10 min at 4 °C in order to remove nuclear contamination, pellets were washed 2 times with DPBS and lysed with commercially available RIPA lysis buffer (Boston BioProducts, Boston, MA, USA, supplemented with protease inhibitors) and saved as nuclear fraction. The supernatant from 690× *g* centrifugation was collected and stabilized with mitochondria stabilization buffer (210 mM mannitol, 70 mM sucrose, 5 mM EDTA, 5 mM Tris, pH 7.6). Mitochondria fraction was obtained by further centrifugation at 3000× *g* for 15 min at 4 °C. The pellet contained mitochondria and was lysed with RIPA lysis buffer, and the supernatant from the last centrifugation was collected and used as the cytosolic fraction. The purity of each fraction was assessed with immunoblotting using mitochondrial (cytochrome c)- and cytosolic (GADPH)-specific markers, respectively.

### 2.10. Immuno-Blotting

Immuno-blotting experiments were performed as preciously described [22]. Proteins were separated by sodium dodecyl sulfate- polyacrylamide gel (SDS-PAGE) and subsequently blotted onto an Immobilon-P polyvinylidene difluoride (PVDF) membrane (Millipore, Burlington, MA, USA). Non-specific signals were minimized with TBST blocking buffer supplemented with 5% milk powder. Primary antibody (caspase 3: 1:1000; caspase 6: 1:1000; cytochrome c: 1:1000, GADPH: 1:10,000, β-actin: 1:10,000) and secondary antibody were subsequently used at a 1:10,000 dilution for both anti-mouse and anti-rabbit horseradish peroxidase, as previously described [22]. Specific protein signals were visualized by chemiluminescence (Merck, Ltd., Kenilworth, NJ, USA) and were detected with ChemiDoc™ XRS+ system (Bio-Rad, Hercules, CA, USA).

### 2.11. Terminal Deoxynucleotidyl Transferase-Mediated dUTP-Biotin Nick End Labeling (TUNEL) Assay

To assess to level of apoptotic cells, paraffin-embedded tissue sections were processed as mentioned above and stained with the TUNEL assay using a DeadEnd^TM^ Fluorometric TUNEL System (Promega, Madison, WI, USA) according to the manufacturer’s instructions with nuclei counterstained with DAPI. Five random images of both the cortex and medulla region of the kidney were taken under 200x magnification using Olympus IX83 microscopy and quantified by CellSens software (Tokyo, Japan). The number of TUNEL-positive cells was divided by the total number of cells in each image to obtain the percentage of TUNEL-positive cells.

### 2.12. Statistical Analyses

Results were expressed as mean ± standard deviation (SD). Comparative studies of means were performed using a one-way analysis of variance (ANOVA) followed by a Kruskal Wallis test. Statistical significance was considered when *p* < 0.05.

## 3. Results

### 3.1. Nanosome-Encapsulated Honokiol Improves Renal Functions of Cisplatin-Injured Animals 

**Figure 1:**
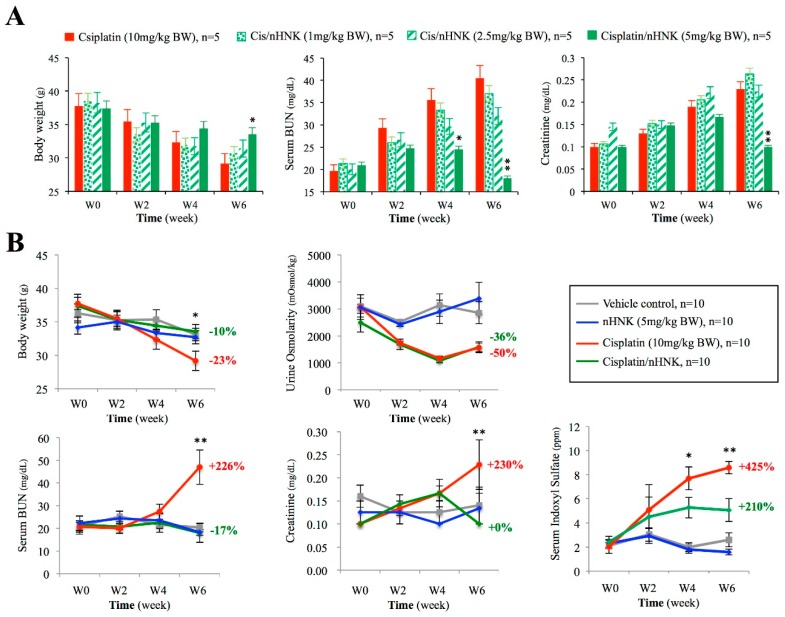
Influences of nanosome Honokiol (nHNK) on selected physiological parameters concerned with renal function. Cisplatin-induced kidney injury and the effects of nanosome honokiol were evaluated by body weight, urine osmolarity, serum blood urea nitrogen (BUN), creatinine, and serum indoxylsulfate. (**A**) Dose-dependent evaluation showed the lowest effective concentration of nHNK to restore cisplatin-induced renal damages and loss of body weight is 5 mg/kg body weight (**B**) Cisplatin administration caused reduced body weight (−23%) and urine osmolarity (−50%) with increased serum BUN (+226%), creatinine (+230%) and serum indoxulsulfate (+425%) in mice at the end of six weeks. Mice in the treatment group (green lines), besides urine osmolarity (–36%), exhibited improved renal functions as evaluated by serum BUN (–17%), creatinine (+0%), and indoxylsulfate (+211%). The percentage shown at each graph represents the relative changes of indicated physiological parameter at the sixth week as compared with its own value at the starting point (0 week). Experiments were carried out with six animals in each group (24 animals in total), and analyses were performed bi-weekly. Data are presented as mean ± standard deviation (SD). Asterisks indicated significant (*p* < 0.05) difference between the cisplatin-injured group (red lines) and the treatment group (in green). Statistics was performed with one-way analysis of variance (ANOVA) followed by a Kruskal Wallis test.

To determine the lowest effective dosage that counteracts cisplatin-induced renal damage, we first applied 0–5 mg/kg B.W. nHNK on mice and evaluated parameters regarding general kidney physiology. As shown in Figure 1A, a time-dependent loss of body weight, increased BUN, and creatinine were observed in cisplatin-treated mice. When cisplatin-injured mice were given different dosages (1, 2.5, 5 mg/kg B.W.) of nHNK, only mice treated with 5 mg/kg B.W. nHNK showed significant improvement on body weight, BUN and creatinine (Figure 1A). Therefore, we considered that 5 mg/kg B.W. nHNK was the lowest effective dosage to be used in our following experiment. When a large-scale experiment was conducted, mice received nHNK alone (blue lines) exhibited no differences on body weight, BUN, urine creatinine and serum indoxylsulfate from those of control animals (grey lines), which indicated that no cytotoxic effect of nHNK was measured in this study (Figure 1B). In contrast, mice which received cisplatin (red lines) exhibited a significant reduction of body weight, urine osmolarity with increased BUN, urine creatinine and serum IS, which indicated severe renal dysfunction in cisplatin-injured animals (Figure 1B). In contrast, mice in the treatment group (green lines) showed no differences on body weight, BUN and serum creatinine when compared with control animals. Although these mice did not reach a full recovery when serum IS was examined, a significant improvement was observed when compared with the cisplatin-injured group (Figure 1B, compare red lines with green lines, significant differences between these two groups are marked with asterisks), which suggested basic kidney functions were improved when nHNK was given to cisplatin-injured mice. Apart from those improved renal physiological parameters, we observed no improvement on urine osmolarity upon nHNK treatment. Although we did not detect proteinuria (data not shown) under our experimental setup, low urine osmolarity measured in the treatment group indicated that cisplatin induced a non-reversible defect on urine concentration ability, but had not yet interfered with the protein filtration function of glomeruli of these mice in our model system. Detailed measurements of all parameters at each time point are summarized in Table 1.

### 3.2. Nanosome-Encapsulated Honokiol Reduces Pathological Changes, Kidney Inflammation and Fibrosis in Cisplatin-Injured Mice

**Figure 2:**
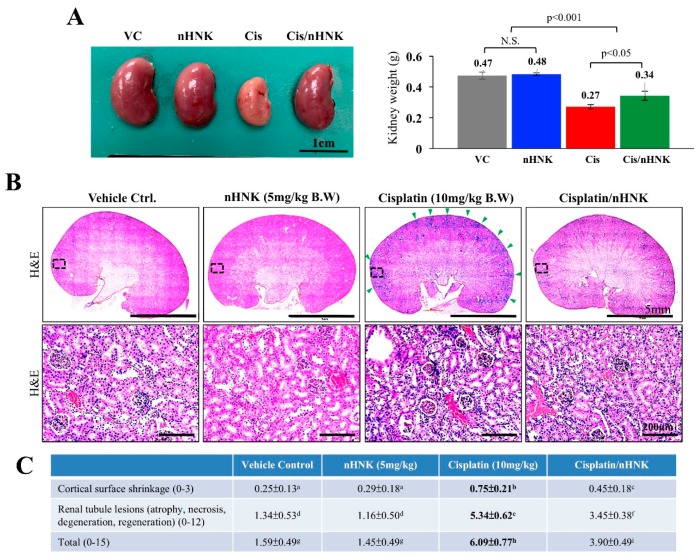
Histological and pathological evaluations of cisplatin-induced damages in kidney. (**A**) Kidneys of cisplatin-injured animals were smaller in size with pale appearance and were significantly lighter than kidneys of other groups. (**B**) Under hematoxylin and eosin (H and E) stain, a significant increase in the infiltration of inflammatory cells around the blood vessels and at the cortex region were observed in cisplatin-injured animals (marked with arrowheads and was enlarged with higher magnification). (**C**) Pathological scoring showed a basic scoring of 1.59 ± 0.49 and 1.45 ± 0.49 in control and nHNK groups while cisplatin-receiving animals scored significantly higher values of 6.09 ± 0.77 for their pathological changes in kidneys, and a significantly reduced pathological scoring of 3.90 ± 0.49 was calculated in the treatment group. Data were presented as mean ± SD, and statistical significance was set to *p* < 0.05 under one-way analysis of variance (ANOVA) followed by a Kruskal Wallis test, a–i indicate statistical difference between groups. Representative images are presented.

**Figure 3:**
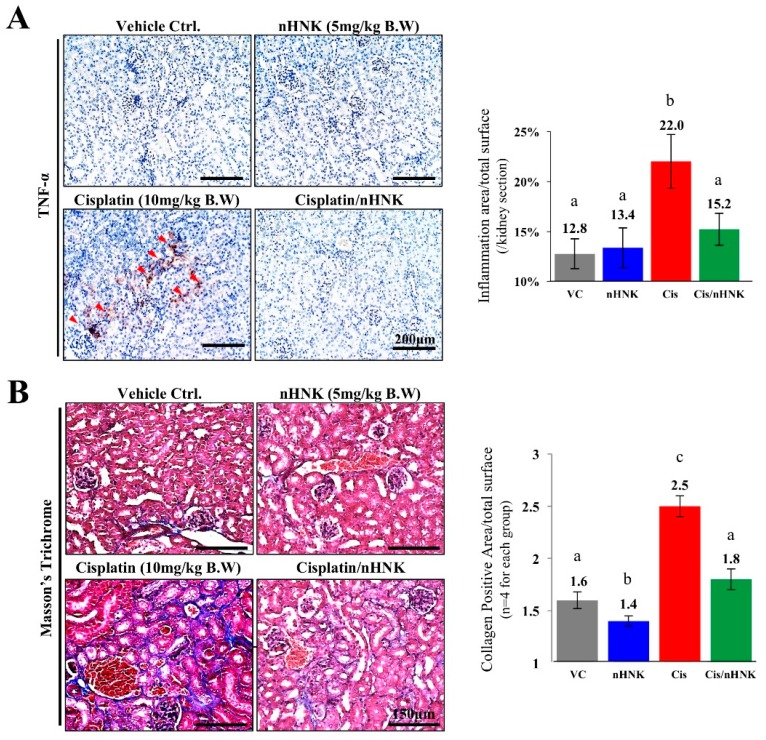
Evaluation of kidney inflammation and collagen deposition. Paraffin-embedded kidney sections were subjected to immunohistochemistry and Masson’s trichrome analyses. (**A**) Pro-inflammatory cytokine tumor necrosis factor-α was detected exclusively in cisplatin-receiving animals (9/10 animals, marked with red arrowheads), while no signals can be detected in other groups. Cisplatin-injured animals exhibited significant higher amounts of inflammatory cell infiltration (22% of total kidney area examined) than control (12.8%), nHNK alone (13.4%) and the treatment group (15.2%). (**B**) Masson’s trichrome staining showed an increased fibrosis, stained in blue (2.5%, +56% when compared with vehicle control) in cisplatin-receiving animals while significantly less collagen fiber deposition was observed in control (1.6%) and treatment (1.8%, +13% when compared with vehicle control) groups. Images presented are representative images, and 10 kidney sections per experimental condition were subjected for evaluation. Data are presented as mean ± SD, and statistical significance was set to *p* < 0.05 under one-way analysis of variance (ANOVA) followed by a Kruskal Wallis test, a–c indicate statistical difference between groups.

As shown in Figure 2A, kidneys of cisplatin-treated animals were smaller in size and had a pale-yellow appearance under gross necropsy examination when compared with kidneys of other groups. Moreover, kidneys of cisplatin-injured animals were significantly lighter (0.27 ± 0.03 g) than those of control (0.47 ± 0.02 g), nHNK alone (0.48 g ± 0.01 g) or treatment (0.34 ± 0.01 g) groups. Under H and E stain, an increase of inflammatory cells around the blood vessels and the cortex region were noticed in cisplatin-injured animals (Figure 2B, indicated with green arrowheads), however, when nHNK was given to cisplatin-injured animals, a reduction of inflammatory cells was observed. In agreement with physiological parameters shown in Figure 1, cisplatin-injured animals received the highest total scores (6.09 ± 0.77) among all groups under pathological evaluation, and mice in the treatment group received significantly lower pathological scores (3.9 ± 0.49) (Figure 2C). Moreover, pathological evaluation indicated that renal tubule lesions was one of the most severely damaged structures in cisplatin-injured mice. To evaluate the severity of inflammations, kidney slides (*n* = 10 for each condition) were subjected to three pathologists for triple-blind quantification. A significant increase in inflammatory area per kidney section was scored in cisplatin-injured animals (22%) while the treatment group showed no differences when compared with control or nHNK alone animals (12.8%, 13.4% and 15.2% for control, nHNK alone and treatment group respectively, Figure 3A). Positive signals for TNF-α were detected exclusively in kidneys of cisplatin-injured mice (9/10 animals showed positive signals), and no signals were detected in other groups, indicating reduced inflammation and inflammatory cytokine, TNF-α, in the treatment group (Figure 3A). Masson’s trichrome stain showed a significantly higher amount of fibrosis in cisplatin-injured animals (Figure 3B, collagen fibers stained in blue). Despite the fact that an increase of fibrosis was also observed in the treatment group, the amount was significantly less when compared with cisplatin-injured animals (Figure 3B). Taken together, our data demonstrated that nHNK attenuated cisplatin-induced renal damages in vivo, as reflected on physiological parameters presented, moreover, reduced inflammation, renal interstitial fibrosis and less severe overall kidney lesions were also observed in nHNK-treated animals.

### 3.3. Nanosome-Encapsulated Honokiol Reduces Cellular Oxidative Damages And Maintains Cellular Localization of Mitochondrial Enzyme Cytochrome C

**Figure 4:**
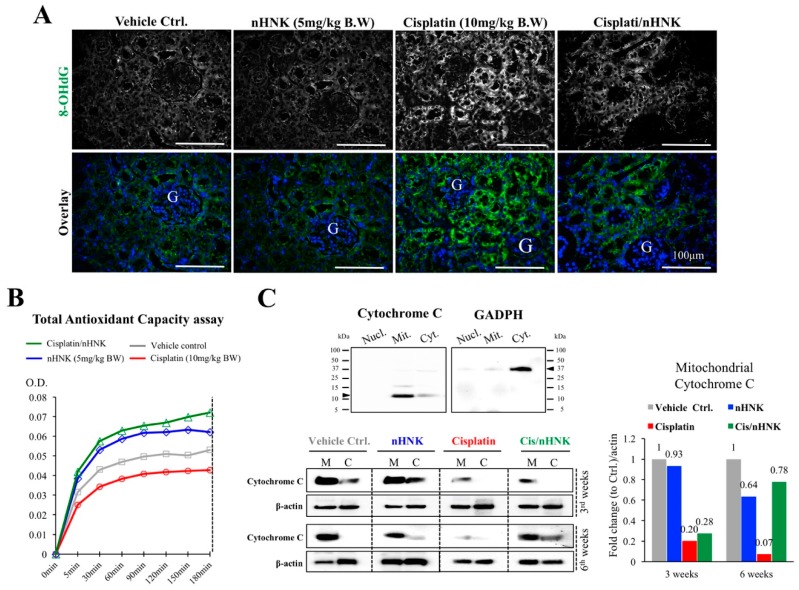
Oxidative stress assessments on the reduction of oxidative damages and improved antioxidant capacity in nHNK-treated animals. (**A**) Paraffin-embedded kidney sections were used, and oxidative stress was assessed with marker protein 8-hydroxyguanosine. Control and nHNK groups showed minimal amounts of 8-OHdG, an intense signal can be detected in the cisplatin-injured animals, and signal intensity was decreased in the treatment group. (**B**) To assess the antioxidant capacity of the kidney, total antioxidant capacity (TAC) assay was carried out. Cisplatin-injured kidney showed the lowest total mitochondria enzyme activity as assessed by TAC assay (red line). Groups in the presence of nHNK (blue and green lines) showed the highest two recording optical density values, which indicated that more active mitochondria enzymes were available in these groups, which suggested that nHNK maintained and facilitated the antioxidation property of the kidney. (**C**) Western-blotting analysis showed the majority of the cytochrome c protein appeared in the mitochondria fraction (M) of control, nHNK alone and the treatment groups with minimal detection in the cytosolic fraction (C). However, a significant reduction of total cytochrome c protein expression in the mitochondria fraction was observed in cisplatin-injured animals.

It is known that cisplatin induces oxidative stress [10,18]. We next examined whether the reduction of kidney pathological changes was related to the improvement of oxidative status of the kidney. As shown in Figure 4A, a minimal amount of OS marker protein 8-OHdG (in green) was observed in control and nHNK groups while an increased signal of 8-OHdG was detected at the proximal tubules of cisplatin-injured animals and an apparent reduction of 8-OHdG signal was observed in the treatment group (Figure 4A). To qualitatively access OS and to evaluate whether this reduced OS was associated with mitochondria antioxidant enzyme activities, we applied TAC assay on whole kidney tissue homogenates. As demonstrated in Figure 4B, a clear reduction on TAC was measured in the cisplatin group (red line) as a lower OD value was recorded from kidney homogenates. Of particular interest is the highest two recordings which were observed in the groups with nHNK (green and blue lines), which suggested that nHNK facilitates cellular antioxidant ability via improving antioxidant-related enzyme activities. We next examined cytochrome c, an inner mitochondria membrane protein responsible for mitochondria redox reaction. Cytochrome c has been shown to initiate caspase-dependent apoptosis upon its release into the cytoplasm [24], we showed in Figure 4C that a pronounced decreased in cytochrome c total protein expression upon cisplatin injury (80% less when compared with control) and a gradual recovery on cytochrome c signal was observed in mitochondria fraction when nHNK was given to the cisplatin-injured animals (Figure 4C, 20% to 28% in three week animals and 7% to 78%, in six week animals when compared with cisplatin-injured with nHNK-treated animals). Moreover, we detected in the cisplatin-injured group, that a minimal amount of cytochrome c was present in both mitochondria (M) and cytosolic (C) fraction, whereas in the control and nHNK groups, the majority of the cytochrome c remains in the mitochondria fraction (Figure 4C), and more importantly, a pronounced recovery on mitochondrial cytochrome c was detected in the mitochondria fraction in the nHNK treatment group, which indicated that cisplatin-induced release of cytochrome c from the mitochondria to cytosol was attenuated by nHNK.

### 3.4. Nanosome-Encapsulated Honokiol Reduces Cisplatin-Induced Apoptosis

**Figure 5:**
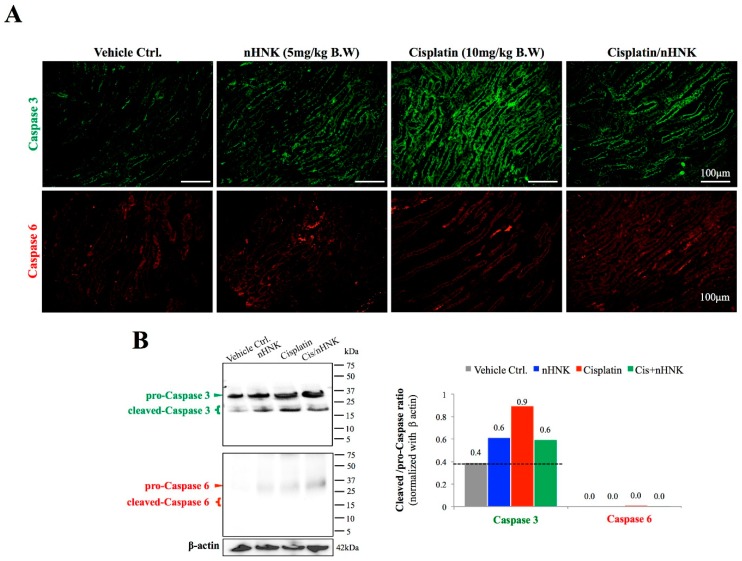
Evaluation of caspase-dependent signaling pathway in cisplatin-injured animals. Caspase 3 and caspase 6 were used to evaluate the activation of cellular apoptosis process. (**A**) A minimal signal on both caspase 3 and caspase 6 was observed in control and nHNK alone groups. An intense caspase 3, but not caspase 6, signal was detected at the collecting duct of cisplatin-injured kidneys, which indicated that cisplatin induced pronounced cellular apoptosis in kidney collecting ducts. A decreased signal on caspase 3 was detected when nHNK was given to cisplatin-injured animals, which demonstrated that nHNK mitigated cisplatin-induced cellular apoptosis. (**B**) Western-blotting analysis showed cisplatin-induced cleavage of pro-caspase 3 enzyme, but these increases were attenuated when nHNK was present. Images presented are representative images of each group.

**Figure 6:**
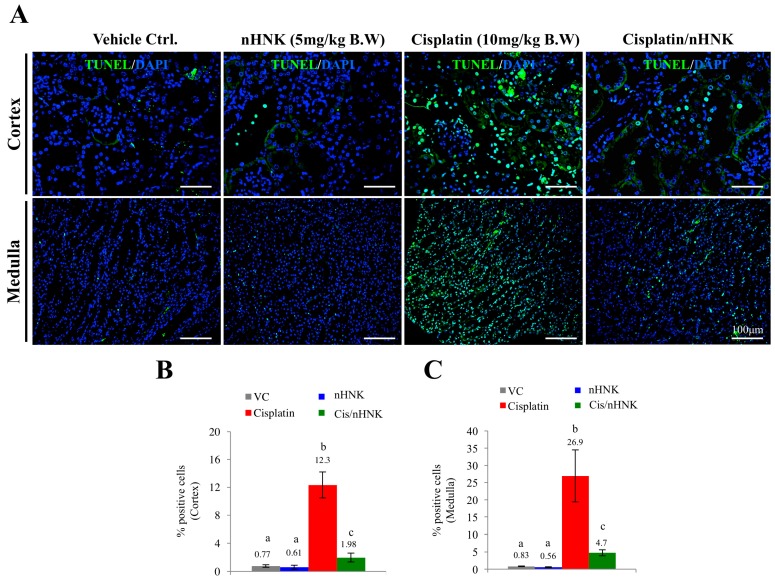
Evaluation of honokiol effects on cellular apoptosis in kidney of cisplatin-injured animals. TUNEL assay was used to evaluate the level of cellular apoptosis. (**A**) In both cortex and medulla, minimal amounts of apoptotic cells were observed in control and nHNK alone groups (less than 1% in these conditions, **B**,**C**). A dramatic increase of apoptotic cells was detected in both the cortex (12.3%, **B**) and medulla (26.9%, **C**) regions of the kidney, however, when nHNK was given to cisplatin-treated animals, significant decreases in apoptotic cells were observed (1.98% and 4.7% in the cortex and medulla regions, respectively).

Damages in mitochondria function with dislocalized cytochrome c trigger intrinsic cell apoptosis. We next examined protein expressions of two apoptosis markers, caspase 3 and caspase 6, as these proteins are known to participate in execution of cellular apoptosis in both human and mouse. In contrast to control and nHNK alone kidneys with minor amounts of caspase 3 being detected, an intense caspase 3 signal was observed at the collecting ducts of cisplatin-injured animals, which indicated that cisplatin-induced apoptosis occurred mostly on the kidney collecting duct under our experimental setup (Figure 5A). An apparent reduction of caspase 3 signal was observed when nHNK was given to cisplatin-injured animals (Figure 5A). Despite the fact that weak signals were also detected for caspase 6 in cisplatin-injured and the treatment groups, no apparent differences between groups were noted. Western-blotting analyses showed an increase (+125%) in active (cleaved, 17 kDa) caspase 3 protein expressions in cisplatin-injured animals when compared with control animals, and in agreement with immunofluorescent assay study, a reduction (−33%) in active caspase 3 protein expression was detected when nHNK was given to cisplatin-injured animals, indicating that nHNK reduced cisplatin-induced caspase 3-associated apoptosis in kidneys (Figure 5B). Despite minor pro-caspase 6 signals being detected, no cleaved caspase 6 could be detected in all groups examined (Figure 5B). DeadEnd Fluorometric TUNEL assay was next used to evaluate the level of apoptotic cells at different regions of the kidney. In agreement with findings from Figure 5, we observed in the cortex and medulla, a minimal TUNEL-positive signal in control and nHNK kidneys (Figure 6A), but a significantly higher proportion of TUNEL-positive cells were counted in cisplatin-injured kidneys (Figure 6B–C, 16-fold increase in the cortex and 32-fold increase in the medulla). More importantly, when nHNK was given to cisplatin-injured animals, pronounced reductions in TUNEL-positive cells were observed in both kidney regions (Figure 6B–C, 6.2-fold reduction in the cortex and 5.8-fold reduction in the medulla).

## 4. Discussion

Liposome encapsulation of lipophilic compounds to improve their solubility and bioavailability for clinical applications has been widely used in the past decades. In this study, we demonstrated that nano-sized liposome-encapsulated polyphenol constituent honokiol efficiently attenuated cisplatin-induced chronic renal damages by reducing kidney inflammation, oxidative stress and caspase 3-associated cellular apoptosis. The reduction of oxidative stress is likely achieved by maintaining cytochrome c at the inner mitochondria membrane and thus improves mitochondria redox ability and mitochondria integrity. The maintenance of cellular mitochondrial functions by nHNK may at least partially account for the reduction of caspase 3-mediated cellular apoptosis and thereafter resulted in the observed counteraction of honokiol on cisplatin-induced pathological damages and the improved renal physiology in vivo. 

Cisplatin is known to cause cell death by forming DNA adducts that lead to cell cycle arrest and cell apoptosis [2]. Although it has been used to effectively treat various cancers [1], its nephrotoxicity is one of the main dose-limiting side effects to prevent its systemic application at full efficacious doses [3,6]. Several studies, including ours, have shown that in addition to DNA damages, the excessive production of ROS and RNS and the inhibition of antioxidant enzyme activities caused by cisplatin are also the main deteriorating factors that lead to cisplatin cytotoxicity [10,19,25,26]. We demonstrated earlier in vitro that cisplatin disorganized cellular localization of E-Cadherin and Occludin, delayed polymerization dynamic of actin and tubulin, and as a consequence, compromised epithelial cell polarity. Moreover, mitochondria total antioxidant capacity was reduced due to an imbalanced mitochondria redox process [19]. In this study, we applied a six week cisplatin-induced chronic renal injury mouse model and showed that nHNK mitigated cisplatin-induced renal damages in vivo. Based on parameters for renal physiology (e.g., BUN, creatinie), the 5 mg/kg B.W. used was the lowest effective concentration that showed significant protective effects among our dosage evaluation studies. It is of particular interest that we observed an improved renal physiology in terms of reduced serum BUN, creatinine and indoxyl sulfate, however, urine osmolarity did not improve after nHNK treatment, suggesting cisplatin caused an irreversible damage on kidney collecting ducts and altered the urine concentration ability. 

Although disruption of urine concentration ability does not always necessarily correlate, at the early phase, to structural abnormality, it can simply be due to the alteration on the function or normal constitutive recycling of water channel molecules (e.g., AQP2). However, in our model system, besides defects on urine concentration ability, we also observed structural abnormality based on general pathological scoring that an increase in renal tubule lesions was detected. It is known that cisplatin tends to accumulate in renal tubules due to the imbalance between cisplatin intake and output transporters at the renal tubule epithelium [9,27]. From our observation, we observed accumulation of 8-OHdG signal at the proximal tubule but not glomeruli of the kidney. Moreover, an increased amount of caspase 3-positive cells were also detected at the collecting duct of the kidney. These observations may account for the irreversible urine concentration ability, but no proteinuria was detected in our model system. In line with recent in vitro and in vivo studies which showed that honokiol exhibited anti-inflammation bioactivity [12,28,29], we observed the reduction of inflammatory cells at the interstitial area with minimal to no detection of TNF-α in the treatment group. All these above-mentioned effects likely resulted in an improved renal physiology in general in cisplatin-injured animals.

In agreement with our earlier in vitro study, in this study, an increased detection of 8-OHdG was observed at the proximal tubules of cisplatin-injured animals, this accumulation of oxidative damage marker protein was not only due to the increase of free radicals caused by cisplatin, but also by the reduction of mitochondria redox ability as the key enzyme cytochrome c, which is known to be responsible for maintaining mitochondria respiratory chain and was significantly reduced upon cisplatin treatment. Many proteins and enzymes (e.g., oxidative phosphorylation, OXPHOS) have been correlated with mitochondria function [30]. In the current study, we aimed to demonstrate the mitochondria integrity and their general antioxidant capacity/ability in our in vivo model, we therefore chose total antioxidant assay (TAC) to obtain functional activity of mitochondria antioxidant capacity as a whole cellular organelle and demonstrated the structural integrity of mitochondria by additional cellular fractionation experiments. We observed higher TAC activities in groups in the presence of nHNK (both nHNK alone and the treatment group), and the results of TAC assays reflect this correspondingly to the cellular localization and the total amount of cytochrome c detected from the Western-blotting analyses. It is known that honokiol can serve as a ROS scavenger [17,18], although we did not measure directly the ROS production from these animals, the reduction of 8-OHdG in the kidney of the treatment group indicated a protective outcome when nHNK was applied. In this study, we did not address whether the increase or decrease of 8-OHdG was accompanied with the changes of lipid oxidation markers, e.g., 4-hydroxynonenal (4HNE) or malondialdehyde (MDA), however, we cannot exclude the possibility that cisplatin also increases lipid oxidation in general, therefore, future study focusing on this specific aspect may improve the understanding of honokiol effects on lipid oxidation and advance mechanistic understanding of honokiol effects.

It is known that both cisplatin and honokiol are anti-cancer compounds, despite no drug–drug interaction ever being demonstrated between cisplatin and honokiol, published literatures supported the fact that a combination of honokiol with cisplatin exhibited a synergic effect on cancer treatment. [31,32,33]. Although the focus of the current study is not on compound interactions nor cancer research, the above-mentioned studies may suggest potential beneficial or synergic anti-tumor effects when used in combination in cisplatin-receiving patients with reduced renal toxicity. Similar to vitamin E and superoxide dismutase (SOD), honokiol is known to be an antioxidant, we showed that both vitamin E and honokiol efficiently reduced cisplatin-induced ROS in vitro, and no significant differences on antioxidation ability between vitamin E and honokiol were observed when compound concentration was below 5 μM (Figure S4). However, it is worth noting that at a higher concentration of 10 μM, honokiol exhibited significantly better antioxidation ability than that of vitamin E at the same concentration (Figure S4), suggesting that, at least under our in vitro experimental setup, honokiol seemed to be a stronger ROS scavenger than vitamin E.

One of the novelties of the current study is to apply nanotechnology on a lipophilic herbal compound to not only counteract cisplatin-induced nephrotoxicity, but also extend the retention longevity, biodistribution and bioavailability of honokiol, and most importantly, improve therapeutic effectiveness by combining liposomal honokiol with cisplatin, as demonstrated in other earlier studies [31,32,33].

There is indeed, a need from the aspects of pharmacology to address in detail, pharmakinetic interactions between cisplatin and honokiol. Nevertheless, based on our data, in vivo protective effects of honokiol are apparent, and the reduction of oxidative damages in nHNK-treated animals are likely due to the combination of: (1) the natural ROS scavenging property of honokiol, (2) the maintenance of inner mitochondria membrane protein cytochrome c at its cellular location, and (3) the maintenance on the total amount of cytochrome c for functional and sufficient redox activity. This speculation is evidenced by the fact that release of cytochrome c into the cytosol leads to the activation of caspase 3-dependent apoptosis [34]. In the current study, we did not observe accumulation or shift of cytochrome c from mitochondria to cytosol, instead, we observed significant reduction of total cytochrome c protein expression, this is likely attributed to the dilution of cytosolic cytochrome c upon our six week experimental time. Nevertheless, we observed gradual recovery of both total and mitochondria cytochrome c upon nHNK treatment. These above-mentioned effects on mitochondria integrity and reduced active caspase 3 likely account for the significant reduction on DNA fragmentation and apoptotic cells detected by TUNEL assay in nHNK-treated animals. One interesting note is that we also observed the emergence of cytochrome C and cleaved caspase 3 in both control and nHNK alone groups, this indicates that repeated IP injection of either liposome or nHNK alone could also cause minor damages, stress on mitochondria, as well as activate cellular apoptosis to a certain extent. However, the level of mitochondria damages and cellular apoptosis in those cases might be low and minor, so that a healthy individual could maintain regular mitochondria function and structure for further recovery. Therefore, with increasing applications on liposome- and nanomaterial-based therapies/approaches, additional care and evaluation on the safety and natural deleterious effect of these biomaterials should be considered.

## 5. Conclusions

In conclusion, we demonstrated in the current study, that nanosuspension-formulated honokiol provides protective effects against cisplatin-induced kidney damages in vivo, and these protective outcomes and the improvement of renal functions were likely resulted from the combined effects of: (1) reduced cellular oxidative stress, (2) restoration of mitochondria function and integrity, and (3) reduced caspase 3-associated apoptosis, all these above-mentioned consequences resulted in the improved renal physiology of cisplatin-treated animals and mitigated cisplatin-induced nephrotocixity. 

## Figures and Tables

**Table 1:** Kidney physiological parameters (value ± S.D.).

Parameters		Week 0	Week 2	Week 4	Week 6
Body weight (g)	G1 Control	36.26 ± 1.41	35.26 ± 1.47	35.34 ± 1.47	33.10 ± 0.93
	G2 nHNK	34.15 ± 1.35	35.03 ± 1.35	33.35 ± 1.45	32.71 ± 1.29
	G3 Cisplatin	37.74 ± 0.93	35.46 ± 1.14	32.35 ± 1.45	29.18 ± 1.46*
	G4 Cis/nHNK	37.39 ± 1.71	35.28 ± 1.24	34.43 ± 0.84	33.55 ± 1.08
Urine Osmolarity (mOsmol)/kg	G1 Control	3100.0 ± 50	2516.7 ± 36	3140.0 ± 407	2856.3 ± 25
	G2 nHNK	3050.0 ± 351	2425.0 ± 70	2891.7 ± 436	3390 ± 590
	G3 Cisplatin	3088.2 ± 456	1735.9 ± 78	1168.1 ± 83	1558.3 ± 183
	G4 Cis/nHNK	2489.4 ± 347	1815.5 ± 198	1087.5 ± 75	1587.5 ± 188
Serum BUN (mg/dl)	G1 Control	20.84 ± 2.49	25.20 ± 2.35	21.6 ± 1.63	20.40 ± 1.91
	G2 nHNK	22.25 ± 3.10	24.25 ± 3.30	23.50 ± 3.90	18.25 ± 1.25
	G3 Cisplatin	20.81 ± 1.79	20.00 ± 2.12	27.33 ± 3.18	47.00 ± 13.8*
	G4 Cis/nHNK	21.50 ± 4.00	20.75 ± 1.25	22.50 ± 4.17	18.00 ± 4.17
Serum creatinine (mg/dl)	G1 Control	0.160 ± 0.02	0.130 ± 0.03	0.130 ± 0.03	0.140 ± 0.04
	G2 nHNK	0.130 ± 0.03	0.130 ± 0.03	0.100 ± 0.00	0.130 ± 0.03
	G3 Cisplatin	0.100 ± 0.00	0.130 ± 0.02	0.170 ± 0.02	0.230 ± 0.05*
	G4 Cis/nHNK	0.100 ± 0.00	0.143 ± 0.02	0.167 ± 0.03	0.100 ± 0.00
Serum indoxyl sulfate (ppm)	G1 Control	2.017 ± 0.27	3.043 ± 0.55	1.988 ± 0.35	2.600 ± 0.58
	G2 nHNK	2.302 ± 0.33	2.911 ± 0.61	1.810 ± 0.32	1.584 ± 0.23
	G3 Cisplatin	2.021 ± 0.56	5.074 ± 2.08	7.694 ± 0.95*	8.591 ± 0.51*
	G4 Cis/nHNK	2.397 ± 0.51	4.491 ± 1.64	5.256 ± 0.85	5.058 ± 0.95

1. Ctrl.: vehicle control (100 μL corn oil); nHNK: 5 mg/kg liposome-encapsulated honokiol alone, IP; Cisplatin: 10mg/kg cisplatin alone, IP; Cis/nHNK: 10 mg/kg cisplatin + 5 mg/kg Honokiol. 2. * indicates the G3 cisplatin group is significantly different (*p* < 0.05) from the G4 (treatment) cisplatin + honokiol group.

## Supplementary Materials

The following are available online at https://www.mdpi.com/2076-3921/8/10/466/s1, Figure S1 Qualitative and quantitative characterizations of liposome-encapsulated honokiol, Figure S2 Establishment of cisplatin-induced chronic kidney injury model, Figure S3 Standardization of serum indoxylsulfate detection by high-performance liquid chromatography in kidney tissues, Figure S4 Measurement and comparisons on the antioxidation ability between vitamin E and honokiol by flow cytometry using membrane-permeable dye 2′, 7′-dichlorofluorescein diacetate (DCFH-DA).

## References

[1] Dasari S., Tchounwou P.B. (2014). Cisplatin in cancer therapy: Molecular mechanisms of action. Eur. J. Pharmacol..

[2] Garcia Sar D., Aguado L., Montes Bayon M., Comendador M.A., Blanco Gonzalez E., Sanz-Medel A., Sierra L.M. (2012). Relationships between cisplatin-induced adducts and DNA strand-breaks, mutation and recombination in vivo in somatic cells of Drosophila melanogaster, under different conditions of nucleotide excision repair. Mutat. Res..

[3] Zhu S., Pabla N., Tang C., He L., Dong Z. (2015). DNA damage response in cisplatin-induced nephrotoxicity. Arch. Toxicol..

[4] Kuhlmann M.K., Burkhardt G., Kohler H. (1997). Insights into potential cellular mechanisms of cisplatin nephrotoxicity and their clinical application. Nephrol. Dial. Transplant..

[5] Ozkok A., Edelstein C.L. (2014). Pathophysiology of cisplatin-induced acute kidney injury. Biomed Res. Int..

[6] Karasawa T., Steyger P.S. (2015). An integrated view of cisplatin-induced nephrotoxicity and ototoxicity. Toxicol. Lett..

[7] Ciarimboli G., Deuster D., Knief A., Sperling M., Holtkamp M., Edemir B., Pavenstädt H., Lanvers-Kaminsky C., am Zehnhoff-Dinnesen A., Schinkel A.H. (2010). Organic Cation Transporter 2 Mediates Cisplatin-Induced Oto- and Nephrotoxicity and Is a Target for Protective Interventions. Am. J. Pathol..

[8] Miller R.P., Tadagavadi R.K., Ramesh G., Reeves W.B. (2010). Mechanisms of Cisplatin Nephrotoxicity. Toxins.

[9] Wagner D.J., Hu T., Wang J. (2016). Polyspecific organic cation transporters and their impact on drug intracellular levels and pharmacodynamics. Pharmacol. Res..

[10] Choi Y.-M., Kim H.-K., Shim W., Anwar M.A., Kwon J.-W., Kwon H.-K., Kim H.J., Jeong H., Kim H.M., Hwang D. (2015). Mechanism of Cisplatin-Induced Cytotoxicity Is Correlated to Impaired Metabolism Due to Mitochondrial ROS Generation. PLoS ONE.

[11] Sureshbabu A., Ryter S.W., Choi M.E. (2015). Oxidative stress and autophagy: Crucial modulators of kidney injury. Redox Biol..

[12] Munroe M.E., Businga T.R., Kline J.N., Bishop G.A. (2010). Anti-inflammatory effects of the neurotransmitter agonist Honokiol in a mouse model of allergic asthma. J. Immunol. (Baltim. Md. 1950).

[13] Sulakhiya K., Kumar P., Gurjar S.S., Barua C.C., Hazarika N.K. (2015). Beneficial effect of honokiol on lipopolysaccharide induced anxiety-like behavior and liver damage in mice. Pharm. Biochem. Behav..

[14] Cheng S., Castillo V., Welty M., Eliaz I., Sliva D. (2016). Honokiol inhibits migration of renal cell carcinoma through activation of RhoA/ROCK/MLC signaling pathway. Int. J. Oncol..

[15] Chiang C.K., Sheu M.L., Lin Y.W., Wu C.T., Yang C.C., Chen M.W., Hung K.Y., Wu K.D., Liu S.H. (2011). Honokiol ameliorates renal fibrosis by inhibiting extracellular matrix and pro-inflammatory factors in vivo and in vitro. Br. J. Pharmacol..

[16] Liu J., Zhang C., Liu Z., Zhang J., Xiang Z., Sun T. (2015). Honokiol downregulates Kruppel-like factor 4 expression, attenuates inflammation, and reduces histopathology after spinal cord injury in rats. Spine.

[17] Shen J.L., Man K.M., Huang P.H., Chen W.C., Chen D.C., Cheng Y.W., Liu P.L., Chou M.C., Chen Y.H. (2010). Honokiol and magnolol as multifunctional antioxidative molecules for dermatologic disorders. Molecules.

[18] Dikalov S., Losik T., Arbiser J.L. (2008). Honokiol is a Potent Scavenger of Superoxide and Peroxyl Radicals. Biochem. Pharmacol..

[19] Wang T.J., Liu H.T., Lai Y.H., Jan T.R., Nomura N., Chang H.W., Chou C.C., Lee Y.J., Tsai P.J. (2018). Honokiol, a Polyphenol Natural Compound, Attenuates Cisplatin-Induced Acute Cytotoxicity in Renal Epithelial Cells Through Cellular Oxidative Stress and Cytoskeleton Modulations. Front. Pharm..

[20] Khushnud T., Mousa S.A. (2013). Potential role of naturally derived polyphenols and their nanotechnology delivery in cancer. Mol. Biotechnol..

[21] Han M., Yu X., Guo Y., Wang Y., Kuang H., Wang X. (2014). Honokiol nanosuspensions: Preparation, increased oral bioavailability and dramatically enhanced biodistribution in the cardio-cerebro-vascular system. Coll. Surf. B Biointerfaces.

[22] Kuo Y.W., Joshi R., Wang T.E., Chang H.W., Li S.H., Hsiao C.N., Tsai P.J. (2017). Identification, characterization and purification of porcine Quiescin Q6-Sulfydryl Oxidase 2 protein. BMC Vet. Res..

[23] Clayton D.A., Shadel G.S. (2014). Isolation of mitochondria from animal tissue. Cold Spring Harb. Protoc..

[24] Elena-Real C.A., Diaz-Quintana A., Gonzalez-Arzola K., Velazquez-Campoy A., Orzaez M., Lopez-Rivas A., Gil-Caballero S., De la Rosa M.A., Diaz-Moreno I. (2018). Cytochrome c speeds up caspase cascade activation by blocking 14-3-3epsilon-dependent Apaf-1 inhibition. Cell Death Dis..

[25] Khynriam D., Prasad S.B. (2002). Changes in glutathione-related enzymes in tumor-bearing mice after cisplatin treatment. Cell Biol. Toxicol..

[26] Yasuyuki S., Takahiro S., Yoshio T. (1992). Mechanism of the increase in lipid peroxide induced by cisplatin in the kidneys of rats. Toxicol. Lett..

[27] Vickers A.E., Rose K., Fisher R., Saulnier M., Sahota P., Bentley P. (2004). Kidney slices of human and rat to characterize cisplatin-induced injury on cellular pathways and morphology. Toxicol. Pathol..

[28] Li N., Xie H., Li L., Wang J., Fang M., Yang N., Lin H. (2014). Effects of honokiol on sepsis-induced acute kidney injury in an experimental model of sepsis in rats. Inflammation.

[29] Chao L.K., Liao P.C., Ho C.L., Wang E.I., Chuang C.C., Chiu H.W., Hung L.B., Hua K.F. (2010). Anti-inflammatory bioactivities of honokiol through inhibition of protein kinase C, mitogen-activated protein kinase, and the NF-kappaB pathway to reduce LPS-induced TNFalpha and NO expression. J. Agric. Food Chem..

[30] Wilkens V., Kohl W., Busch K. (2013). Restricted diffusion of OXPHOS complexes in dynamic mitochondria delays their exchange between cristae and engenders a transitory mosaic distribution. J. Cell Sci..

[31] Cheng N., Xia T., Han Y., He Q.J., Zhao R., Ma J.R. (2011). Synergistic antitumor effects of liposomal honokiol combined with cisplatin in colon cancer models. Oncol. Lett..

[32] Jiang Q.Q., Fan L.Y., Yang G.L., Guo W.H., Hou W.L., Chen L.J., Wei Y.Q. (2008). Improved therapeutic effectiveness by combining liposomal honokiol with cisplatin in lung cancer model. BMC Cancer.

[33] Liu Y., Chen L., He X., Fan L., Yang G., Chen X., Lin X., Du L., Li Z., Ye H. (2008). Enhancement of therapeutic effectiveness by combining liposomal honokiol with cisplatin in ovarian carcinoma. Int. J. Gynecol. Cancer.

[34] Shukla K.K., Mahdi A.A., Rajender S. (2012). Apoptosis, spermatogenesis and male infertility. Front. Biosci. (Elite Ed).

