# Supplementary material for: Nanoparticulated Honokiol Mitigates Cisplatin-Induced Chronic Kidney Injury by Maintaining Mitochondria Antioxidant Capacity and Reducing Caspase 3-Associated Cellular Apoptosis
Source: Antioxidants (Basel). 2019 Oct 9;8(10):466. doi: 10.3390/antiox8100466 (PMC6826708; doi:10.3390/antiox8100466)
Supplement: Supplementary file 1 [file antioxidants-08-00466-s001.pdf]

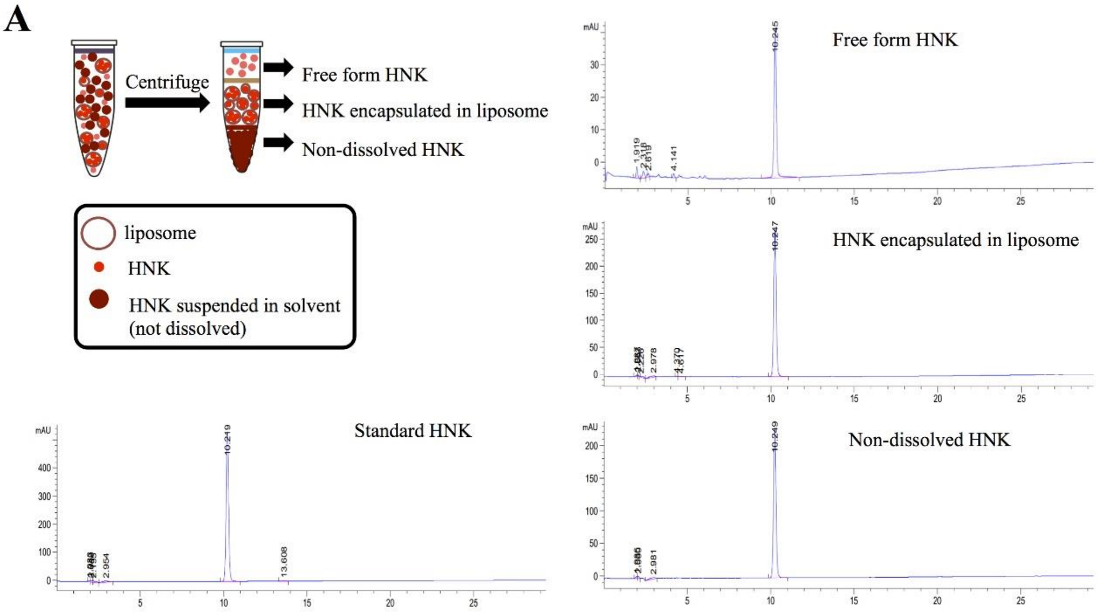

B

	Area (254 nm)	Concentration (ppm)	amount in 1 mL sample (mg)	
Free HNK	445.09	0.0081	0.005±0.000	
HNK in liposome	2740.11	0.0501	0.780±0.033	1.343±0.056
Non-dissolved HNK	2366.66	0.0433	0.557±0.023	

Encapsulation efficiency= 0.780/1.343*100%= **58.1±2.44%**

Figure 1. Qualitative and quantitative characterizations of liposome-encapsulated honokiol.

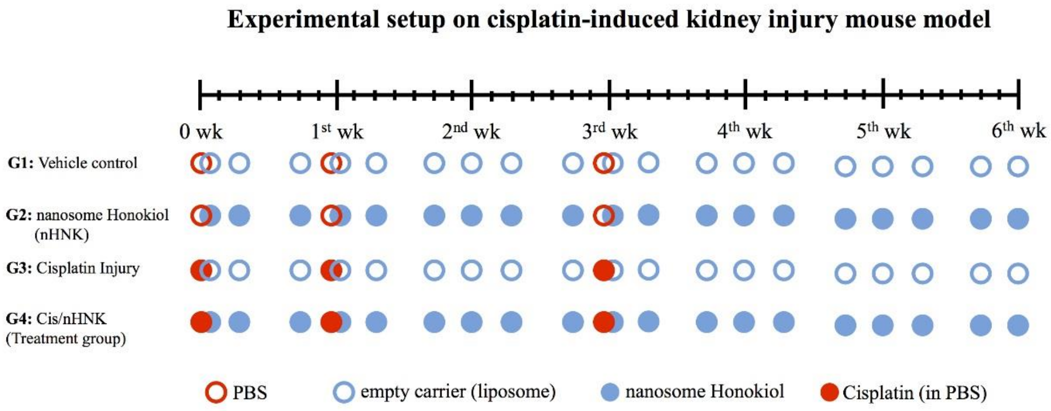

Figure 2. Establishment of cisplatin-induced chronic kidney injury model

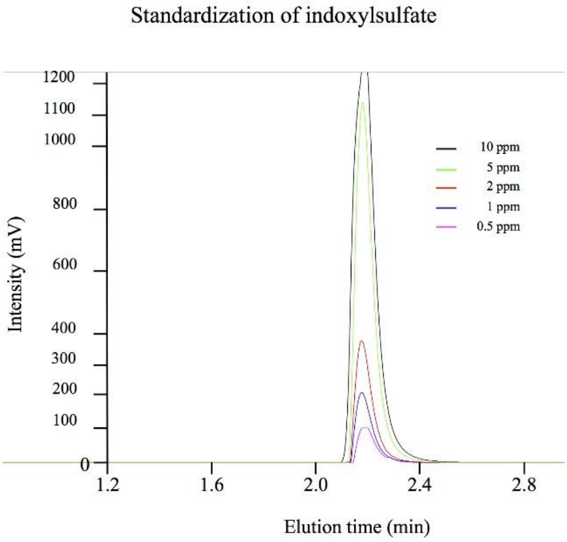

Figure 3. Standardization of serum indoxylsulfate detection by high-performance liquid chromatography in kidney tissues

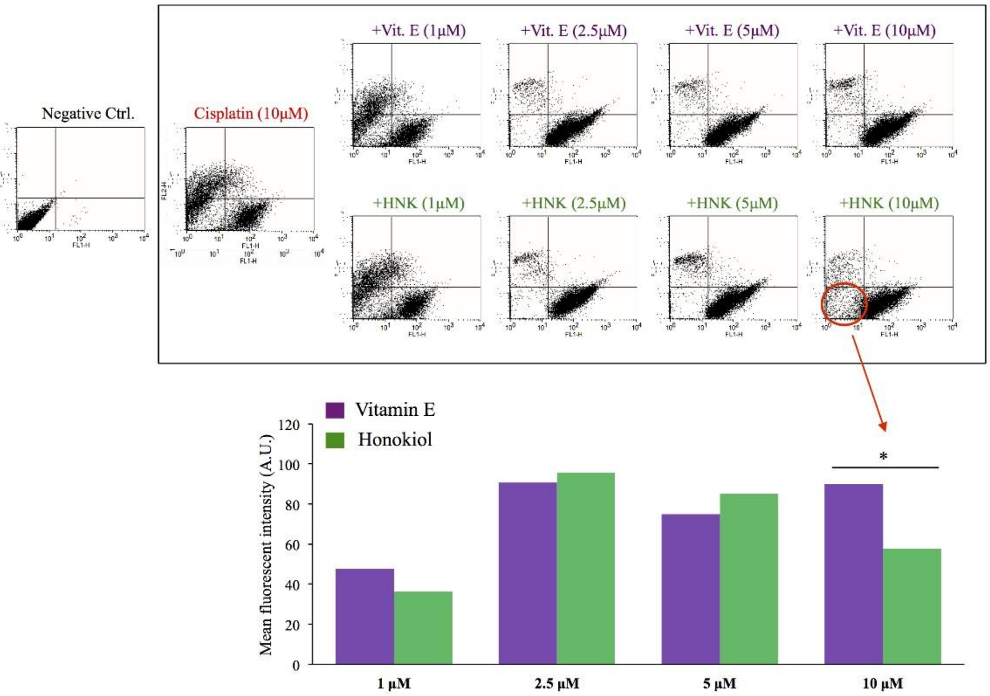

Figure 4. Measurement and comparisons on the antioxidation ability between vitamin E and honokiol by flow cytometry using membrane-permeable dye 2′, 7′-dichlorofluorescein diacetate (DCFH-DA).
